# Edited Magnetic Resonance Spectroscopy Detects an Age-Related Decline in Nonhuman Primate Brain GABA Levels

**DOI:** 10.1155/2016/6523909

**Published:** 2016-08-31

**Authors:** Xuanzi He, Bang-Bon Koo, Ronald J. Killiany

**Affiliations:** ^1^Memory and Aging Center, Department of Neurology, University of California San Francisco, San Francisco, CA 94158, USA; ^2^Department of Anatomy and Neurobiology, Boston University School of Medicine, 620 Albany St., Boston, MA 02118, USA

## Abstract

Recent research had shown a correlation between aging and decreasing Gamma-aminobutyric acid (GABA), the primary inhibitory neurotransmitter in the brain. However, how GABA level varies with age in the medial portion of the brain has not yet been studied. The purpose of this study was to investigate the GABA level variation with age focusing on the posterior cingulate cortex, which is the “core hub” of the default mode network. In this study, 14 monkeys between 4 and 21 years were recruited, and MEGA-PRESS MRS was performed to measure GABA levels, in order to explore a potential link between aging and GABA. Our results showed that a correlation between age and GABA+/Creatine ratio was at the edge of significance (*r* = −0.523, *p* = 0.081). There was also a near-significant trend between gray matter/white matter ratio and the GABA+/Creatine ratio (*r* = −0.518, *p* = 0.0848). Meanwhile, the correlation between age and grey matter showed no significance (*r* = −0.028, *p* = 0.93). Therefore, age and gray matter/white matter ratio account for different part of *R*-squared (adjusted *R*-squared = 0.5187) as independent variables for predicting GABA levels. Adjusted *R*-squared is about 0.5 for two independent variables. These findings suggest that there is internal neurochemical variation of GABA levels in the nonhuman primates associated with normal aging and structural brain decline.

## 1. Introduction

As we age, the brain undergoes gradual functional and cognitive declines; aging is related to neurochemical alterations in the central nervous system and cortical disruptions. Γ-Aminobutyric acid (GABA) is the primary inhibitory neurotransmitter in the brain and plays an integral role in metabolism and neuronal activity. GABA dysfunction is implicated diverse physiological and neurological diseases, such as schizophrenia [1], ADHD [2]. Various studies report cognitive deficiencies associated with aging, like motor decision speed [3] and the tendency for cognitive failures [4]. Aging has also been demonstrated to be related to cortical network disruption in structures such as precuneus, retrosplenial, and posterior cingulate cortices [5]. Moreover, previous research has shown aging influenced default mode network (DMN), which is an essential neuronal network underlying brain functions [6]. The most relevant and recent research [7] used MEGA-PRESS for GABA detection localized on human frontal and parietal cortex. Their results demonstrated age-related GABA+ decline trend in those two cortical areas. In the current study, we focused on the posterior cingulate cortex, which is the “core hub” of default mode network [8]. We hypothesized that the increasing age would associate with the decreasing GABA+ concentration in the posterior cingulate cortex of nonhuman primates.

Although some previous studies reported age-related GABA declines [9–11], comprehensive research investigating age-related changes in GABA concentrations has not been conducted yet to the best of our knowledge. One of the advantages of using nonhuman primates as the subject is that the monkeys never develop Alzheimer disease, which means that they are the ideal subject for normal aging research.

**Figure 1:**
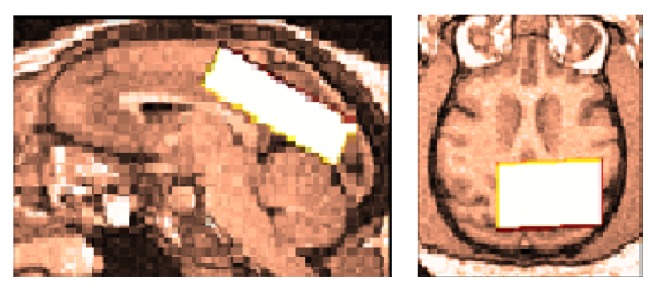
MRS VOI position: the volume of interest was placed in the posterior cortical cortex, without including the splenium of the corpus callosum. The voxel location is indicated by the white rectangle shown on axial and sagittal views.

GABA detection is comparatively challenging as its concentration is an order of magnitude lower than that of other metabolites. Therefore, using standard single-voxel techniques is not practical due to the overlap of GABA and other metabolites mainly Creatine (Figure 1), whose amount is much larger than GABA around 3.0 ppm. Meanwhile, another vital characteristic of MRS spectrum, spin-spin coupling (J-coupling), provided the crucial clue for disentangling the GABA signal from other metabolites. The so-called multiplets in MR spectrum stem from the neighboring spin interaction. Although the broader footprint and lower peak intensity caused by J-coupling lead to the difficulty of GABA detection, the coupling of GABA signal around 3.0 ppm with the signals at 1.9 ppm differentiates itself from the other primary signals at 3.0 ppm, which are not coupled with the signal at 1.9 ppm. The edited GABA detection took advantage of this interaction by applying a frequency-selective (edited) pulse on 1.9 ppm, influencing the GABA signal on 3.0 ppm indirectly, while the other signals on 3.0 ppm are not affected.

Consequently, the difference between edited pulse ON at 1.9 ppm and edited pulse OFF version will illustrate merely the signals influenced by the edited ON pulse. The resulting edited spectrum filters out majority signal at 3.0 ppm, which is not coupling with the signal at 1.9 ppm. Among many of the spectral editing methods, MEscher-GArwood Point RESolved Spectroscopy (MEGA-PRESS) is widely used for GABA quantification due to its simplicity of implementation. As the name implies, MEGA-PRESS adds two frequency-selective editing pulses on the PRESS single-voxel MRS experiment.

## 2. Methods

### 2.1. Subjects

Sixteen aging Rhesus monkeys (*Macaca mulatta*) that were part of ongoing studies of healthy aging at Boston University in June to November of 2012 were imaged after anesthetizing. All monkeys (age: range 4–21, mean = 15.5 ± 6.07 years) had complete medical and birth records and were in good health.

### 2.2. Ethical Statement

This project followed the rules of Animal Care Program of Boston University, Boston, MA.

### 2.3. MRI and MRS Acquisition

All subjects were scanned on a 3 T scanner (Philips “Achieva” TX Best, The Netherlands) using an eight-channel phased array head coil receiver. T1-weighted three-dimensional Fast Field Echo images were obtained with the following parameters: TR = 250 ms, TE = 3.163 ms; slice thickness = 0.6 mm; matrix 256 × 256 pixels; field of view (FOV) = 220 × 220 mm; and flip angle = 90°. The volume of interest (VOI) with a size of 10 × 30 × 35 mm^3^ was chosen in the medial brain, focusing on the posterior cingulate cortex. The median sagittal plane acted as a reference slice for voxel localization: the VOI was arranged to keep away from the ventricles and skull, as they might influence the result of MRS. The region of interest was set to be aligned with the shape of the corpus callosum and superior to the splenium of the corpus callosum. The medial plane was positioned large enough to cover the PPC area.

### 2.4. MRS Setting

The GABA concentration was measured using an MEGA-PRESS sequence [12], and the parameters are as the following: TR/TE = 1500/68 ms; 16 ms editing pulses alternating at 1.9 and 7.5 ppm to separate the GABA molecule from other chemicals. The deficiency of standard single-voxel techniques had been improved by utilizing an editing pulse (EDIT-ON) at GABA spins at 1.9 ppm to separate the GABA signal from the other metabolites like Creatine at 3.0 ppm, while EDIT-OFF referred to the pulse applied to somewhere else other than 3.0 ppm. The difference between the EDIT-ON and EDIT-OFF spectra yields those peaks affected by the editing pulses. Because the contribution of macromolecules (MM) could not be excluded in every respect as restrained by the fitting limitations, in the following part of this manuscript, the signal detected at 3 ppm is labeled as GABA+ instead of GABA, indicating the potential occurrence of these other compounds. GABA+ and Glx concentration were quantified from the MEGA-PRESS specialized tool Gannet v2.0 [2], which fit the GABA+ model into Gaussian distribution. GABA+ qualification was calculated with the area under the curve, and GABA+ levels were gauged via GABA+/Creatine ratio. Creatine (Cr) performs an adequate reference because of the common location of original of the GABA+ and Cr signals, which comes from the EDIT-OFF MEGA-PRESS spectra.

### 2.5. VOI Localization and VOI Coregistration

VOI was placed in posterior cingulate cortex, in a relatively large area as shown in Figure 1. The voxel has been put in the PCC with an aim to encompass as much of the localized activity as possible, but while also avoiding the scalp/muscle/fatty tissue. Given the need to achieve sufficient signal-to-noise and also some degree of regional specificity, placement of MRS volume of interest can be a difficulty.

Volume of interest (VOI) was coregistered to the structural images via developed Matlab tool called* mrsvoi* to confirm the accuracy of VOI creation; this method could create more reliable mask comparing Gannet's Coregister tool. The grey matter (GM) fraction was defined as the GM volume to the white matter (WM) volume in VOI. Other gray matter indexes are the ratio between GM volume and the whole brain volume and the ratio between GM volume and the volume of VOI.

### 2.6. The Brain Components Segmentation

In general, we followed the BET-FLIRT-FAST pipeline from FSL package (Oxford University, Oxford, UK) [13]. BET (Brain Extraction Tool) erases all the areas outside of brain from a whole brain image, such as T1 or T2 models [14]. FLIRT (FMRIB's Linear Image Registration Tool) is the automatic tool for linear (affine) brain image registration [13]. The second step, FLIRT, is not required for human brain imaging processing, but it is an essential step for monkey brain imaging processing before FAST, due to the variability of the monkey brain. FLIRT could improve the accuracy of image template registration and then enhance the following processing step. Lastly, FAST (FMRIB's Automated Segmentation Tool) divides a structural brain image into different tissue types (grey matter, white matter, CSF, etc.), while also correcting for bias field or RF inhomogeneities [15]. To be more specific, we did the imaging analysis as the following: The T1-weighted brain images were skull-stripped via BET (Brain Extraction Tool) from FSL package [13]. We opted to use the Rhesus Macaque Atlases from Wisconsin ADRC imaging group because of already having an extensive group of users and also used 3 mm FWHM as smoothing for creating each monkey's templates [16]. The structural T1 and grey-prior templates from this template set were firstly rotated according to the orientation of the monkey samples; then the brain image registration tool FLIRT (FMRIB's Linear Image Registration Tool) was used to produce customized templates considering the variation of monkeys' brain. At last, the T1-weighted monkey brain images were segmented as gray matter (GM), white matter (WM), or cerebrospinal fluid (CSF) by FAST (FMRIB's Automated Segmentation Tool) [15] from FSL package, while standardizing to the customized template produced from FLIRT.

### 2.7. GABA+ Measurement Qualification

**Figure 2:**
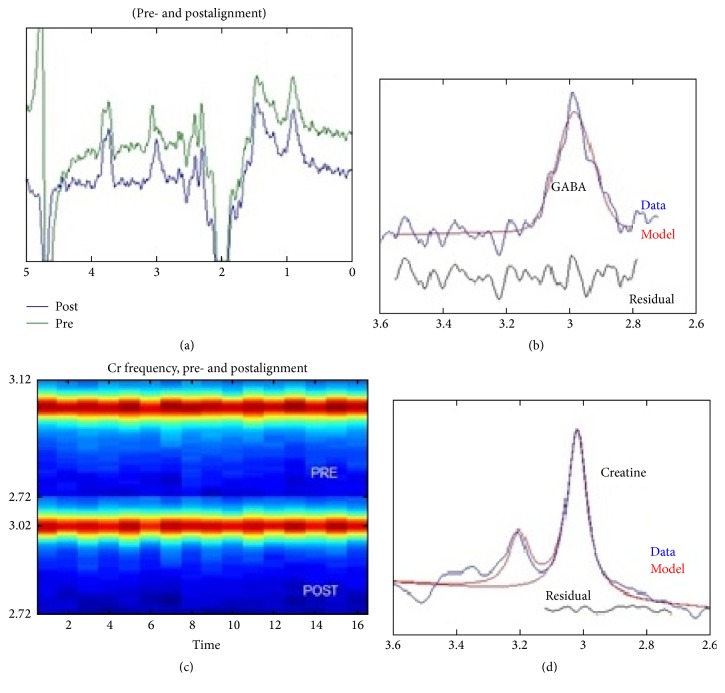
The original GABA+ spectrum editing results: (a) the processed GABA-edited difference spectrum (EDIT-OFF − EDIT-ON = DIFF), which is the primary output of the* GannetLoad* module. Green spectrum is the one* before *frequency and phase correction, while the blue line is the one* after *frequency and phase correction. (b) Model fitting of GABA+ and Creatine spectrum peak: this plot represents the GABA+ signal modeling (*GannetLoad *output). The blue line is the actual GABA-edited spectrum while the red one is the model of best fit (using a simple Gaussian model by default). The residual is the black curve below the modeling plot. (c) Cr signal variation through the whole experiment; the *y*-axis represents the frequency of Cr signal (ppm); the intensity registers with a color scale, so the Cr signal appears as “hot” stripe through the plot. The lower half line should be more evenly horizontal compared with the upper half strip due to the frequency and phase correction. (d) The bottom panel is the Cr signal from the OFF spectrum.

One of the examples of the GABA+ spectrum is shown in Figure 2. The quality control of Gannet is standard deviation of residual, and the baseline of references modeling served as the vital foundation of success modeling GABA+ concentration. Three monkeys' GABA+ spectra were excluded from further statistical analysis due to low model fitting as the Fit Error (Cr) excesses 45% in those cases (the definition of Fit Error is the standard deviation of the residuals expressed as a percentage of the signal height). The remaining monkeys' GABA+ concentration ranged from 0.06 to 0.1 (mean = 0.08, sd = 0.01).

### 2.8. Statistical Analysis

The relationships between variables (e.g., age; gray matter in VOI; GABA+/Cr) were examined via Pearson Product Correlation (*p* < 0.05, uncorrected). All group differences were tested on unpaired 2-tailed *t*-tests (*p* < 0.05, uncorrected). Analyses were performed with *R* (*R* version 3.0.3).

## 3. Results

### 3.1. The Outlier Examination

Two outlier subjects were excluded from further analysis based on Weisberg *t*-test, which is a robust outlier test at small values. The GABA+/Creatine ratio of one monkey is much higher than the others (0.14 compared to mean of all other monkeys: 0.0784); another monkey had an aberrant value of gray matter (2.26*E* + 04 compared to an average of all other monkeys: 18953). Besides the two outliers, one monkey has two records of GABA+ excitation: the second time scan was 15 days later than the first scan. We chose the version from the same terminal as other monkies (longitudinal terminal instead of DSI terminal).

### 3.2. Group Difference Comparisons

**Figure 3:**
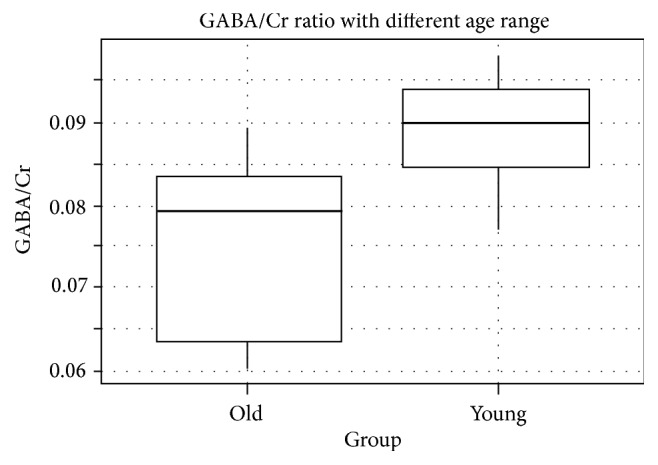
Box plot of GABA levels grouped by age. ns: not significant; *t*-test. Abbreviations: GABA, G-aminobutyric acid; Cr, Creatine.

We grouped the monkeys into two cohorts based on their age (young: 7.5 (±) 2.38; old: 19.5 (±) 0.76). The VOI placement and spectral fit for GABA and Glx were not different between two groups. The GABA/Cr ratio difference between old group (0.08 (±) 0.01) and young group (0.09 (±) 0.01) had a clear tendency to significance (*p* = 0.05834, *t*-test, Figure 3). On the other hand, the amount of gray matter in VOI was not that different between two groups (*p* = 0.9074, *t*-test). Besides GABA+, we also measured Glx (Glutamate + Glutamine) since it contains Glutamate, which is the dominant excitatory neurotransmitter. The Glx level was not different between two cohorts (*p* = 0.25, *t*-test).

### 3.3. Correlations and Linear Regression Models

**Figure 4:**
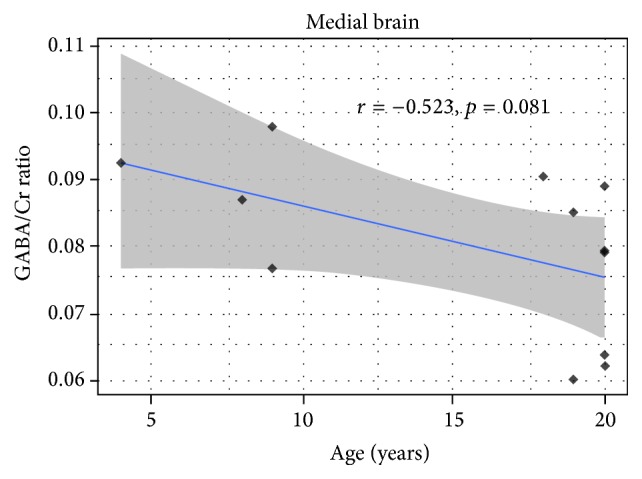
The correlation between GABA/Cr ratio and age.

**Figure 5:**
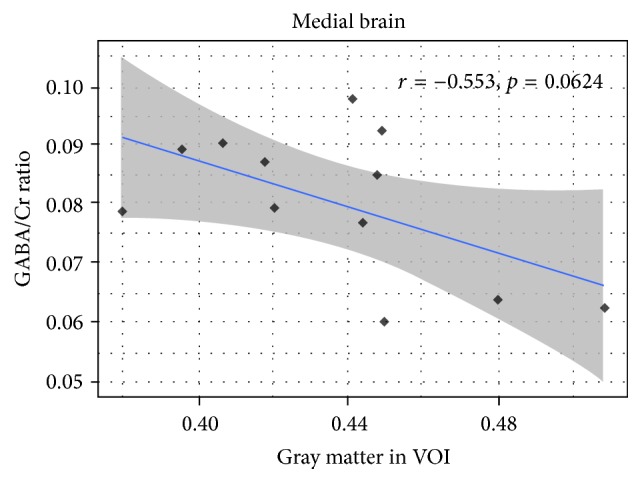
The correlation between GABA/Cr ratio and VOI GM.

**Figure 6:**
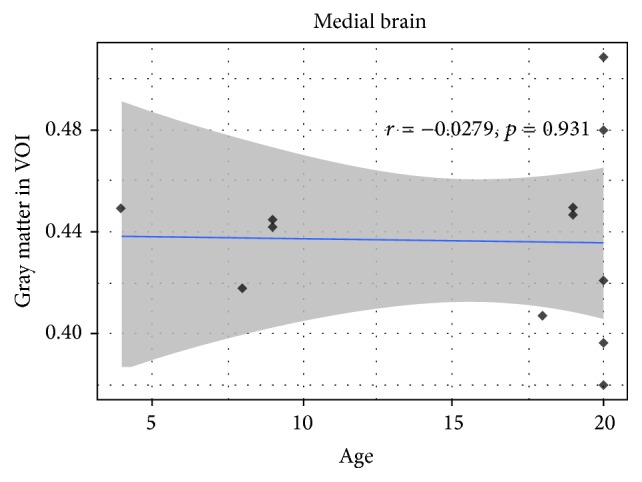
The correlation between age and GM/VOI.

After examining the Pearson Product Correlation between all the factors, GABA+/Creatine ratio, respectively, reached a considerable trend toward significance with age (*r* = −0.523, *p* = 0.081 in Figure 4) and gray matter in VOI voxel (*r* = −0.553, *p* = 0.06 in Figure 5). Meanwhile, the correlation between age and GM/VOI showed no significance (*r* = −0.028, *p* = 0.93 in Figure 6). The relationship between those variables implies age and gray matter are related to GABA/Creatine ratio but not related to each other. This result suggested a general linear model using both age and gray matter as independent variables for predicting GABA/Creatine ratio (Table 1). The adjusted *R*-squared is about 0.5 for two independent variables, while satisfying all the hypotheses of linear regression.

The correlation between Glx/Creatine and GABA+/Creatine was not significant (*r* = 0.0196, *p* = 0.9516), as well as Glx/Creatine and age (*r* = −0.273, *p* = 0.3906). We also utilized GABA+/Glx ratio as a reference, yet GABA+/Glx was not significantly correlated with any essential factors, such as GM/VOI and age.

## 4. Discussions

Using Creatine as a normalizing factor for GABA quantification could cause confounding effect on the result. One of the recent meta-analysis studies [17] reported that Creatine increases with age, which implies in our case GABA+/Cr ratio would be influenced by the Creatine variation. Since no unsuppressed water spectra were collected, it was not possible to normalize GABA+ to the unsuppressed water signal. For clarifying this point, we examined the Pearson Product Correlation between GABA+ and age (*r* = −0.53, *p* = 0.07275). The correlation coefficient did not change that much compared to the correlation between age and GABA+/Cr ratio; at least in our case Creatine was not that influential as it might be. Moreover, Creatine was not related to the increasing age (*r* = −0.21, *p* = 0.45). Glx level was not significantly related to age either (*r* = 0.32, *p* = 0.3091).

With regard to the question of whether the main result (the trend towards a correlation between GABA+/Cr and age) is driven by GABA+ differences with aging or by Cr changes with age, the lack of a significant correlation between Glx/Cr and age provides indication that the reported correlation between GABA+/Cr and age is real (and driven by GABA+ rather than Creatine).

The discrepancy between our results and Haga et al.'s may come from the following: (1) The subject pool in Haga et al. study was the aging human brains while our subjects are nonhuman primate brains. (2) Most data of this meta-analysis came from the frontal region, while in this study the volume of interest concentrated on the posterior cingulate cortex. After all, it is difficult to compare GABA+/Cr ratios between this and the previous studies, as different measurements and parameters, as well as VOI setting, were utilized.

The most substantial finding of this study was a decreasing trend of GABA+ levels with increasing age and increasing gray matter-white matter ratio in the medial brain region of healthy monkeys ranging from 4 to 21 years old. Moreover, age and gray matter tissue fraction do not correlate with each other, which means the variation of age is related to GABA+ concentration in medial portions of the brain. The group comparison results also support this idea.

G-Aminobutyric acid (GABA), the primary inhibitory neurotransmitter in neural system, unexpectedly had not frequently been studied due to the measurement limitation. Recently, various reliable MRS protocols rendered the possibility to measure the GABA in brain reliably [18].

Many researchers suggest that the GABA reduction might be the cause of various cognitive disorders such as memory loss, one of the aging syndromes [19, 20]. However, to the best of our knowledge, only a handful of MRS studies have scrutinized direct age-related changes in GABA concentrations, and most of them were human and other animal models [7]. Although some studies found no correlation between GABA concentration and aging [21], they suffered from the low statistical power, and those findings were not the primary purpose of those studies. Other studies found there was little age dependency of GABA+ [22], however, but they concentrated on the anterior cingulate cortex instead of the posterior cingulate cortex. Many studies realized the GABA concentration in ACC associated with default mode network (DMN), which implied the connection with posterior cingulate cortex (PCC), the core hub of DMN. However, so far the current study is the first research concentrating on posterior cingulate cortex (PCC) as a more direct measurement of the relation between GABA and DMN.

The major inhibitory interneurons are engaged in GABA and therefore presumably the GABA reduction with aging implies* GABAergic neuron* loss during aging [23]. This study offers little insight, as GABAergic neuronal detection is likely not to be applicable on a general brain segmentation level. Another possibility is* GABAergic neuron* dysfunction; if it is due to dysfunction, either GABA synthesis itself or enzymatic deficiency in Glutamate-Glutamine cycling may be involved.

Aging gives rise to degeneration of brain functions such as learning and memory. Aging has also been illustrated to be related to various brain structural and functional changes. The posterior cingulate cortex (PCC) became the focus of aging research as it serves as the central node for DMN (especially in aging condition). Previous studies indicated metabolic reductions in PCC in early Alzheimer's disease [24]. Moreover, the crucial role of PCC had been showed by previous PET study that the metabolic activity in the posterior cingulate cortex is higher than any other cortical areas in resting-state functional MRI [25]. Additionally, the discriminating high inherent signal alternation appeared compared to other general DMN regions in low-frequency signal exposure [26]. Although the importance of the PCC for aging DMN has been well explored, to date, the relationship between GABA+ concentration and the PCC has not been explored on nonhuman primates. Our results are not overly surprising that decline of GABA+ concentration in the PCC associates with aging based on the previous studies implying this possibility.

## 5. Limitation

Our study suffers from several limitations. One of the most well-known deficiencies of MEGA-PRESS is that macromolecule signals could not be excluded from the editing pulse method and then the GABA signals are merged with macromolecule signals. For our study, the pure GABA detection is technically impossible. Another issue from the methodological aspect is that relatively large voxel size (3 × 3 × 3 cm^3^) is required and the specific region information might be missing. Lastly, the protocol of this study did not contain nonsuppressed water scans; as a result, we could only use metabolite as internal standard rather than using water as a reference. In sum, the results of this study suggest brain GABA+/Cr ratio decreases with age and GM fraction in the medial brain, focusing on the posterior cingulate cortex. Further studies may be able to associate aging and GABA concentration based on DMN functions and aid in the development of new clinical outcomes.

## Figures and Tables

**Table 1:** A general linear model uses both age and *GM/VOI* as independent variables for predicting GABA+ level (multiple regression analysis (*N* = 12), dependent variable: GABA+/creatine ratio).

Independent variables	Estimate	Std. error (S.E.)	*t* value	Pr(>|*t*|)
(Intercept)	0.1777298	0.0321091	5.535	*p* < 0.001
Age	−0.0011562	0.0004191	−2.759	0.022156
GM/VOI	−0.1837662	0.0713761	−2.575	0.029960

Residual standard error: 0.008431 on 9 degrees of freedom.

Multiple *R*-squared: 0.6062, adjusted *R*-squared: 0.5187.

*F*-statistic: 6.927 on 2 and 9 DF, *p* value: 0.01509.

## References

[1] Kegeles L. S., Mao X., Stanford A. D. (2012). Elevated prefrontal cortex gamma-aminobutyric acid and glutamate-glutamine levels in schizophrenia measured in vivo with proton magnetic resonance spectroscopy. *Archives of General Psychiatry*.

[2] Edden R. A. E., Puts N. A. J., Harris A. D., Barker P. B., Evans C. J. (2014). Gannet: a batch-processing tool for the quantitative analysis of gamma-aminobutyric acid-edited MR spectroscopy spectra. *Journal of Magnetic Resonance Imaging*.

[3] Sumner P., Edden R. A. E., Bompas A., Evans C. J., Singh K. D. (2010). More GABA, less distraction: a neurochemical predictor of motor decision speed. *Nature Neuroscience*.

[4] Sandberg K., Blicher J. U., Dong M. Y., Rees G., Near J., Kanai R. (2014). Occipital GABA correlates with cognitive failures in daily life. *NeuroImage*.

[5] Buckner R. L. (2004). Memory and executive function in aging and AD: multiple factors that cause decline and reserve factors that compensate. *Neuron*.

[6] Buckner R. L., Andrews-Hanna J. R., Schacter D. L. (2008). The brain's default network: anatomy, function, and relevance to disease. *Annals of the New York Academy of Sciences*.

[7] Gao F., Edden R. A. E., Li M. (2013). Edited magnetic resonance spectroscopy detects an age-related decline in brain GABA levels. *NeuroImage*.

[8] Fransson P., Marrelec G. (2008). The precuneus/posterior cingulate cortex plays a pivotal role in the default mode network: evidence from a partial correlation network analysis. *NeuroImage*.

[9] Leventhal A. G., Wang Y., Pu M., Zhou Y., Ma Y. (2003). GABA and its agonists improved visual cortical function in senescent monkeys. *Science*.

[10] Hua T., Kao C., Sun Q., Li X., Zhou Y. (2008). Decreased proportion of GABA neurons accompanies age-related degradation of neuronal function in cat striate cortex. *Brain Research Bulletin*.

[11] Stanley E. M., Fadel J. R., Mott D. D. (2012). Interneuron loss reduces dendritic inhibition and GABA release in hippocampus of aged rats. *Neurobiology of Aging*.

[12] Mescher M., Merkle H., Kirsch J., Garwood M., Gruetter R. (1998). Simultaneous *in vivo* spectral editing and water suppression. *NMR in Biomedicine*.

[13] Jenkinson M., Smith S. (2001). A global optimisation method for robust affine registration of brain images. *Medical Image Analysis*.

[14] Jenkinson M., Pechaud M., Smith S. BET2: MR-based estimation of brain, skull and scalp surfaces.

[15] Zhang Y., Brady M., Smith S. (2001). Segmentation of brain MR images through a hidden Markov random field model and the expectation-maximization algorithm. *IEEE Transactions on Medical Imaging*.

[16] McLaren D. G., Kosmatka K. J., Oakes T. R. (2009). A population-average MRI-based atlas collection of the rhesus macaque. *NeuroImage*.

[17] Haga K. K., Khor Y. P., Farrall A., Wardlaw J. M. (2009). A systematic review of brain metabolite changes, measured with 1H magnetic resonance spectroscopy, in healthy aging. *Neurobiology of Aging*.

[18] Mullins P. G., McGonigle D. J., O'Gorman R. L. (2014). Current practice in the use of MEGA-PRESS spectroscopy for the detection of GABA. *NeuroImage*.

[19] Schuler V., Lüscher C., Blanchet C. (2001). Epilepsy, hyperalgesia, impaired memory, and loss of pre- and postsynaptic GABA_B_ responses in mice lacking GABA_B(1)_. *Neuron*.

[20] Riese F., Gietl A., Zölch N. (2015). Posterior cingulate *γ*-aminobutyric acid and glutamate/glutamine are reduced in amnestic mild cognitive impairment and are unrelated to amyloid deposition and apolipoprotein E genotype. *Neurobiology of Aging*.

[21] Goddard A. W., Mason G. F., Almai A. (2001). Reductions in occipital cortex GABA levels in panic disorder detected with 1H-magnetic resonance spectroscopy. *Archives of General Psychiatry*.

[22] Aufhaus E., Weber-Fahr W., Sack M. (2013). Absence of changes in GABA concentrations with age and gender in the human anterior cingulate cortex: a MEGA-PRESS study with symmetric editing pulse frequencies for macromolecule suppression. *Magnetic Resonance in Medicine*.

[23] Kelsom C., Lu W. (2013). Development and specification of GABAergic cortical interneurons. *Cell and Bioscience*.

[24] Minoshima S., Giordani B., Berent S., Frey K. A., Foster N. L., Kuhl D. E. (1997). Metabolic reduction in the posterior cingulate cortex in very early Alzheimer's disease. *Annals of Neurology*.

[25] Gusnard D. A., Raichle M. E. (2001). Searching for a baseline: functional imaging and the resting human brain. *Nature Reviews Neuroscience*.

[26] Fransson P. (2005). Spontaneous low-frequency BOLD signal fluctuations: an fMRI investigation of the resting-state default mode of brain function hypothesis. *Human Brain Mapping*.

